# Caspase-3 Is Transiently Activated without Cell Death during Early Antigen Driven Expansion of CD8^+^ T Cells *In Vivo*

**DOI:** 10.1371/journal.pone.0015328

**Published:** 2010-12-22

**Authors:** Scott McComb, Rebecca Mulligan, Subash Sad

**Affiliations:** NRC-Institute for Biological Sciences, Department of Biochemistry, Microbiology and Immunology, University of Ottawa, Ottawa, Canada; Federal University of São Paulo, Brazil

## Abstract

**Background:**

CD8^+^ T cell responses develop rapidly during infection and are swiftly reduced during contraction, wherein >90% of primed CD8^+^ T cells are eliminated. The role of apoptotic mechanisms in controlling this rapid proliferation and contraction of CD8^+^ T cells remains unclear. Surprisingly, evidence has shown non-apoptotic activation of caspase-3 to occur during *in vitro* T-cell proliferation, but the relevance of these mechanisms to *in vivo* CD8^+^ T cell responses has yet to be examined.

**Methods and Findings:**

We have evaluated the activity of caspase-3, a key downstream inducer of apoptosis, throughout the entirety of a CD8^+^ T cell response. We utilized two infection models that differ in the intensity, onset and duration of antigen-presentation and inflammation. Expression of cleaved caspase-3 in antigen specific CD8^+^ T cells was coupled to the timing and strength of antigen presentation in lymphoid organs. We also observed coordinated activation of additional canonical apoptotic markers, including phosphatidylserine exposure. Limiting dilution analysis directly showed that in the presence of IL7, very little cell death occurred in both caspase-3^hi^ and caspase-3^low^ CD8^+^ T cells. The expression of active caspase-3 peaked before effector phenotype (CD62L^low^) CD8^+^ T cells emerged, and was undetectable in effector-phenotype cells. In addition, OVA-specific CD8^+^ cells remained active caspase-3^low^ throughout the contraction phase.

**Conclusions:**

Our results specifically implicate antigen and not inflammation in driving activation of apoptotic mechanisms without cell death in proliferating CD8^+^ T cells. Furthermore, the contraction of CD8^+^ T cell response following expansion is likely not mediated by the key downstream apoptosis inducer, caspase-3.

## Introduction

Antigen presenting cells (APC) activate rare antigen specific CD8^+^ T cells, and induce their clonal expansion (up to 10,000 fold) within 7 days [1]. Rapid expansion is usually followed by a contraction phase wherein ∼95% of the primed cells are diminished from primary lymphoid tissue [2], [3]. Programmed mechanisms of cell death (apoptosis) would seem to be an obvious fit in the role of eliminating antigen specific cells during contraction [4]. Indeed, death receptors, signaling mechanisms, and pro-apoptotic mediators have been implicated in the removal of activated cells after an immune response [5]–[8]. Conversely, studies examining the importance of apoptotic mechanisms in the immune response have also revealed that apoptotic mediators can be associated with T cell activation, but not death in some *in vitro* models [9], [10].

The complex roles of apoptotic mechanisms in the T cell response are exemplified by the function of the Fas receptor. While mutation of the Fas receptor or its ligand leads to serious lymphoproliferative disorders in both mouse and human [11], the mutation of Fas or loss of its signaling proteins (eg FADD or caspase-8) also results in perplexing defects in antigen induced T cell proliferation [12]–[15]. The importance of caspases in T cell activation is reiterated by defective T cell activation observed in humans lacking functional caspase-8 [16]. Furthermore, chemical inhibitors of caspases have been shown to inhibit T cell activation [17].

More generally, a paradoxical connection between apoptotic mechanisms and cell proliferation is rapidly gaining acceptance [10], [18], [19]. Similarly to caspase-3, it has been observed that another classical marker for apoptosis, phosphatidylserine (PS) exposure, can occur in proliferating CD8^+^ T cells [20]. The programmed death marker (PD-1) has also been shown to be upregulated during CD8^+^ T cell activation. Despite the mounting evidence for a role of apoptotic mechanisms in T cell proliferation, little work has been done to investigate their activation during an *in vivo* T cell response. In addition, the possibility that significant cell death is occurring in parallel with expansion has not been examined. It also remains unclear whether these mechanisms are induced during the contraction phase of the CD8^+^ T cell response.

Through examination of apoptotic markers during the entirety of the CD8^+^ T cell response, insight can be gained into the link between the signaling networks of immune stimulation and cell death. In this report, we have used an antibody and a fluorogenic substrate which specifically detect active caspase-3 to analyze the level of active caspase-3 in antigen specific CD8^+^ T cells throughout the *in vivo* response. We also examine the level of PS exposure and PD-1 expression. We have tracked these markers in OVA specific CD8^+^ T cells as they respond to a common antigen, expressed by two divergent pathogens. CD8^+^ T cell response to the highly immunogenic bacterial pathogen *Listeria monocytogenes* expressing OVA (LM-OVA) was compared with poorly immunogenic, *Salmonella* typhimurium, also expressing OVA (ST-OVA). By simultaneously examining the levels of apoptotic and T cell activation markers, we reveal that antigen-presentation, but not inflammation, induces actively proliferating CD8^+^ T cells to take on an apoptotic like phenotype without cell death. Thus, our results support a model in which caspase-3 is induced by antigenic stimulation and acts atypically during CD8^+^ T cell proliferation. Furthermore, because active caspase-3 remained low throughout contraction, this indicates that the removal of antigen specific CD8^+^ T cells following a response occurs through caspase-3 independent mechanisms.

## Results

### Active Caspase-3 is up-regulated during T cell activation *in vitro*

**Figure 1:**
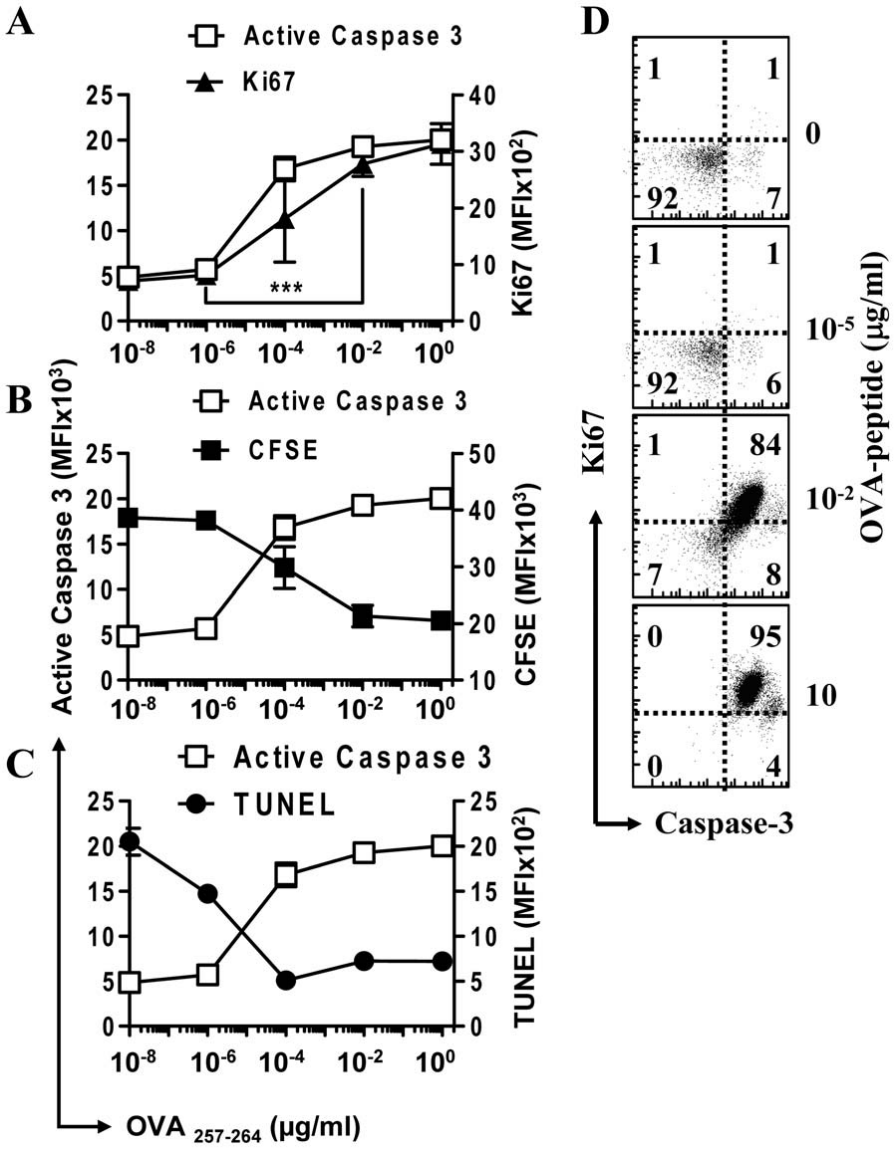
Significant increase in active caspase-3 during T cell activation correlates directly with an increase in proliferation. A single cell suspension of OT1 splenocytes, some of which were first CFSE stained, were placed in culture with varying concentrations of SIINFEKL peptide for 48 hours and analyzed by intracellular flow cytometry (n≥3). Cells were stained with anti-CD8 antibody and OVA-tetramer, then fixed and permeabilized before staining with anti- active caspase-3 antibody and Ki67. Graphs show the mean fluorescence intensity (MFI) of CD8^+^ OVA-tetramer^+^ gated cells. (A) MFI of active caspase-3 versus Ki67 show a high amount of linear correlation in their expression levels (P<0.05). There is a significant increase in the expression of both caspase-3 and Ki67 from 10^−8^ to 10^−2^ µg/mL OVA (P<0.005). (B) MFI of CFSE versus active caspase-3, inverse correlation was found to be significant (P<0.01). (C) MFI of TUNEL stain versus active caspase-3, inverse correlation was found to be significant (P<0.01). (D) Scatterplots show the relative expression of active caspase-3 versus Ki67 for gated CD8^+^ cells in splenocyte cultures treated for 48 hours with varying amounts of SIINFEKL peptide as shown. Data shown is representative for two repeated experiments performed in triplicate.

OVA-specific CD8^+^ TCR transgenic mouse (OT-1) splenocytes were placed in culture with various concentrations of OVA peptide (SIINFEKL). OVA specific CD8^+^ T cells began proliferating within 24 hours of initial stimulation. Intracellular staining was performed using fluorescently labeled antibodies. Those OT-1 CD8^+^ T cells that were stimulated with an amount of antigen greater then about 0.1 nM (10^−4^ µg/ml) showed active proliferation, as identified by high expression of the active cell cycle marker Ki67 (Fig. 1A). The proliferative capacity of these cells was further confirmed by the loss of CFSE staining after activation (Fig. 1B). Coordinated with an increase in proliferation, we observed a significant increase in the level of active caspase-3 in the CD8^+^ population as the antigen levels increased from 10^−8^ to 10^−2^ µg/ml (P<0.005, Fig. 1). Co-staining revealed direct correlation between caspase-3 cleavage and cell proliferation (Ki67^hi^) within the CD8^+^ population (Fig 1D).

**Figure 2:**
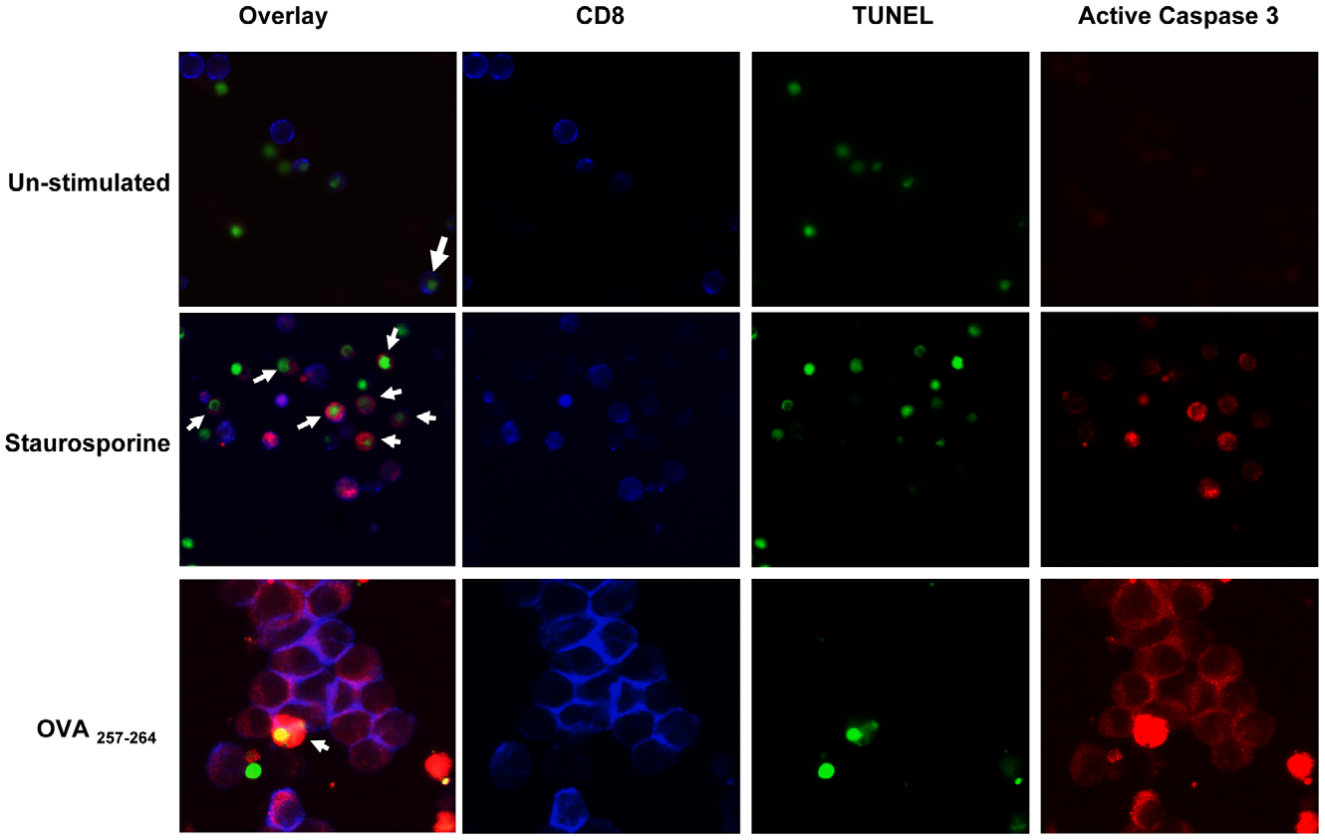
Active caspase-3 in antigen activated T cells is expressed in live proliferating cells. OT1 spleen cells were stimulated in vitro for 48 hours with varying amounts of antigen. Apoptotic control cells were treated with 1 µg/ml staurospaurine. Cells were then stained with anti-CD8 antibody and fixed and permeabilized before TUNEL and caspase-3 staining. Cells were mounted on slides and examinated by fluorescence microscopy. Images show TUNEL staining (green), and active caspase-3 (red) in CD8 labeled T cells (blue). The images are representative of 3 repeated examinations for each differentially stimulated culture. Arrows shown identify cells with positive staining for both caspase-3 and TUNEL.

### Activated CD8^+^ T cells remain viable despite high expression of active Caspase-3

To investigate whether caspase-3 activation is leading to cell death, we next examined the viability of OVA stimulated cells *in vitro*. The viability of proliferating CD8^+^ cells was directly assessed by terminal dUTP nick end labeling (TUNEL) for DNA cleavage. In the absence of any peptide stimulation or growth factors, cells became TUNEL^+^ at later time periods indicating progression to death (Fig. 1C). Stimulation of cells with OVA-peptide resulted in significant reduction of the overall level of DNA cleavage, indicating that stimulated cells were not progressing to apoptotic cell death. Despite the high level of active caspase-3 present in actively proliferating cells there was little indication of increased cell death as determined by TUNEL staining (Fig. 1C). This shows a surprising inverse correlation between the level of active caspase-3 and DNA cleavage as antigenic stimulation of the CD8^+^ T cells increases.

The reduced overall TUNEL staining in *in vitro* activated cultures does not preclude the possibility that a significant level of cell death is occurring in caspase-3^hi^ cells, with a concurrent vigorous outgrowth of active caspase-3^low^ cells. To address this, OT1 spleen cells were activated *in vitro* with LM-OVA. After 18 h, bacteria were removed by gentamicin treatment and OT-1 cells (very low numbers) cultured with addition of cytokine, IL-7. The data indicate that proliferating caspase 3^hi^ CD8^+^ T cells do not progress to cell death (Figure S1). Cells were seeded at such low numbers in order to avoid the statistical probability that a small proliferative subpopulation of cells could account for the majority of the culture while significant cell death is occurring in the majority of cells.

To further confirm cell viability, cells were co-stained directly for CD8, active caspase-3 and DNA cleavage. OT-1 cells were first left un-stimulated, stimulated with OVA-peptide, or with staurosporine (inducer of apoptosis). After 2 days in culture, cells were plated, stained, and examined by fluorescent microscopy. Un-stimulated CD8^+^ T cells showed minimal active caspase-3 expression (Fig. 2), while those treated with staurosporine showed elevated active caspase-3 expression. In the staurosporine treated cells, active caspase-3 was associated with TUNEL in approximately half of active caspase-3 positive cells. In contrast to unstimulated or apoptotic cells, antigen stimulated OT-1 cells grew significantly in size and showed elevated active caspase-3 expression without a significant number of TUNEL^+^ cells (Fig. 2). We observe approximately 5% of cells which showed active caspase-3 to also be positive for TUNEL staining. Cells that were found to be TUNEL^+^/caspase-3^+^ in the OVA-peptide stimulated cultures did not appear to have CD8 expression. Therefore, these results further confirm that caspase-3 is induced during T cell activation, but caspase3^hi^ cells do not progress rapidly to cell death.

### Upregulation of Caspase-3 occurs during early activation of CD8^+^ T cells *in vivo*

**Figure 3:**
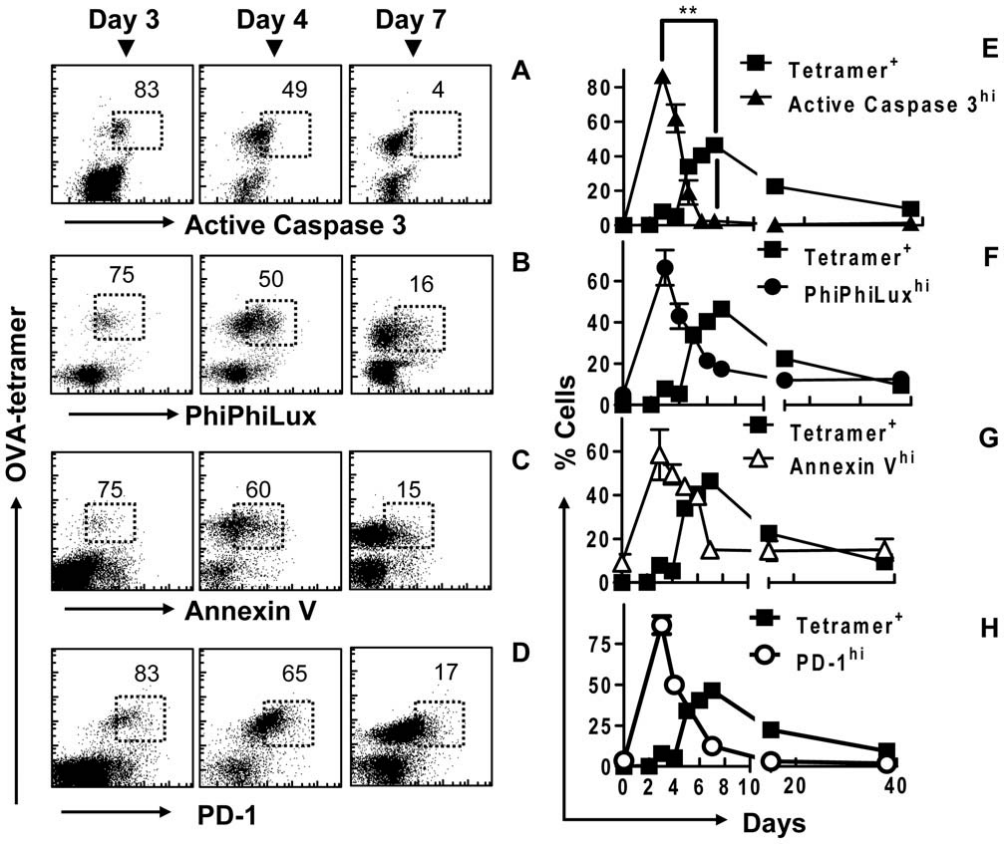
Rapidly proliferating CD8^+^ T cells have an apoptotic like phenotype *in vivo*. C57BL6/J mice were injected with 10^4^ OT1 cells and infected with 10^4^ LM-OVA. At various time points mice were sacrificed and splenic cells were stained and analyzed by FACS. Scatterplots show OVA-tetramer binding versus (A) active caspase, (B) PhiPhiLux cleavage by caspase-3 (PPL), (C) Annexin V binding and (d) PD-1, in cells over days 3, 4 and 7 of LM-OVA infection. The % of OVA-tetramer^+^ CD8^+^ cells expressing a high level of (E▴) caspase-3, (F•) PPL cleavage, (G Δ) Annexin V binding, and (H ○) PD-1 are shown over the course of an acute OVA specific CD8^+^ T cell response (▪). *The decrease in apoptotic staining between day 3 and 7 shows significant linear correlation (by Pearson analysis) between active caspase-3 antibody staining and (E) PPL cleavage activity, (F) annexin V binding and (G) PD-1 expression (P<0.05). **Change in active caspase-3 staining is inversely proportional to an increase in OVA-tetramer specific cells (P<0.001, n =  3 mice/timepoint).

We next evaluated the temporal expression of active caspase-3 during CD8^+^ T cell activation *in vivo*. OVA-specific CD8^+^ T cells were also examined for a range of cell markers, associated with apoptosis and activation, throughout the course of the immune response. At day 3 of LM-OVA infection, >80% of OVA specific CD8^+^ T cells showed highly elevated active caspase-3 expression (Fig. 3A, E). The percentage of OVA-specific CD8^+^ T cells expressing active caspase-3 decreased progressively as responding cells approached the peak of response at day 7 post-infection and remained at a basal level subsequently. Increased caspase-3 activity was confirmed by staining cells with the fluorogenic caspase-3 substrate PhiPhiLux (PPL), which showed similar results (Fig. 3B, F). Cells consistently showed basal levels of active caspase-3 beyond day 40. In addition, upon re-challenge with LM-OVA, OVA-specific CD8^+^ T cells showed rapid and highly transient caspase-3 activation, consistent with secondary expansion of a memory population (data not shown).

Transfer of high numbers of TCR transgenic cells into hosts has been shown to hasten the onset of contraction [21]. To avoid this, we have used small numbers of OT-1 transgenic cells in our adoptive transfer model, which does not alter the programming or contraction of OVA-specific CD8^+^ T cells significantly. Even without adoptive transfer of OT1 cells, proliferating OVA-specific CD8^+^ T cells expressed high levels of caspase 3, which was absent during the contraction phase (Figure S2).

### Active Caspase-3 is accompanied by moderate upregulation of other apoptotic markers

**Figure 4:**
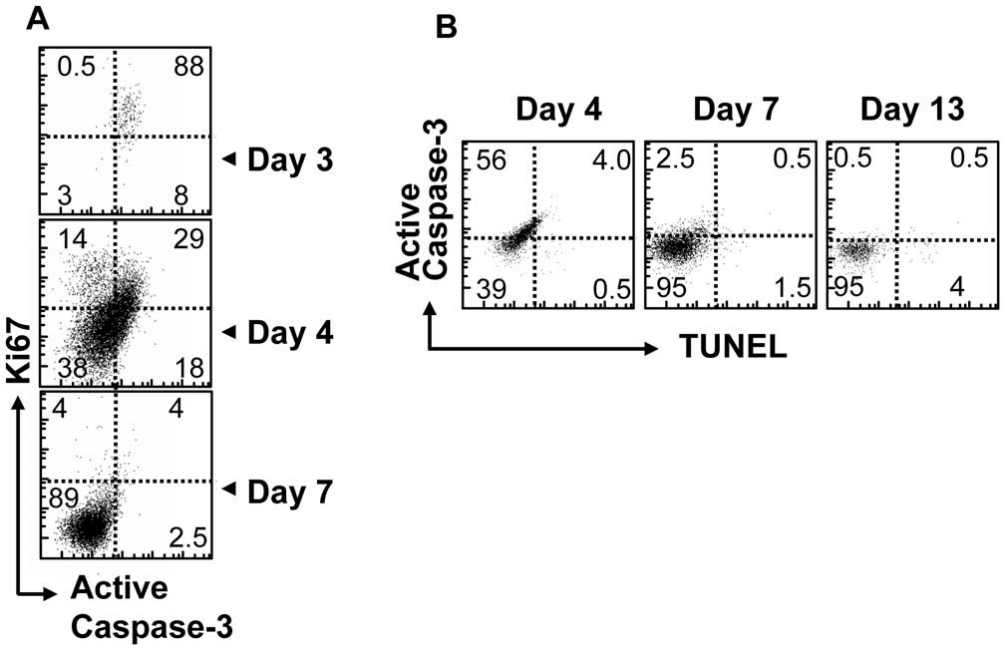
Actively proliferating cells *in vivo* co-express markers for apoptosis and proliferation with little apparent cell death. C57BL/6 mice were parked with 10^4^ OT1 cells and challenged with 10^4^ LM-OVA. Splenic cells were obtained from infected C57BL/6 mice at various time points after infection and stained for CD8 and OVA-tatramer binding as well (A) proliferation marker Ki67 vs. active caspase-3. (B) Cells were also co-stained for DNA cleavage (TUNEL) vs. active caspase-3 expression.

We next evaluated other common markers of apoptosis. Phosphatidylserine (PS) is normally only exposed on the surface of apoptotic cells [22]. Thus, we measured PS exposure using fluorescent annexin V. The upregulation of caspase-3 was accompanied by elevated PS externalization (Fig. 3C, G). It should be noted that when apoptosis was induced by staurosporine, the level of annexin V binding (MFI) in activated cells was determined to be higher than that observed in the proliferating cells (data not shown). At day 3 post-infection the majority of antigen specific CD8^+^ T cells showed that PS externalization increased to an intermediate level, which fell rapidly to basal levels by the peak of the CD8^+^ T cell response at day 7. Programmed death receptor 1 (PD-1) was originally identified as an early marker for cell death [23], thus we also examined its correlation with active caspase-3 during CD8^+^ activation. As with caspase-3 and annexin V binding, OVA-specific CD8^+^ T cells expressed high levels of PD-1 early on, and by day 7 the expression of PD-1 was at basal levels (Fig. 3D, H). Finally, we noted direct correlation between Ki-67 versus caspase-3 expression *in vivo* (Fig. 4A). Collectively, these results indicate that activated OVA specific CD8^+^ T cells have an apoptotic-like phenotype during their early expansion phase, while the cell number continues to increase. However, by the time the numbers of OVA-specific CD8^+^ T cells peak and contraction begins, the expression of these apoptotic mediators is reduced to baseline levels.

### Cells with elevated caspase-3 expression do not progress to cell death *in vivo*

The apparent apoptotic phenotype observed in actively proliferating CD8^+^ T cells *in vivo* required a careful assessment of their viability. Firstly, we examined cell viability directly at various time-points after infection by examining the terminal deoxynucleotidyl transferase dUTP nick end labeling (TUNEL) and active caspase-3 co-staining in antigen specific cells (Fig. 4B). We observed a minimal increase in TUNEL staining associated with active caspase-3 upregulation early in CD8^+^ T-cell response (day 3). It should be noted that some level of increased TUNEL staining can be associated with proliferating cells. We also allowed up to 24 hours *ex vivo* for possible cell death and DNA cleavage to proceed before TUNEL staining, and observed similar results (Figure S3).

**Figure 5:**
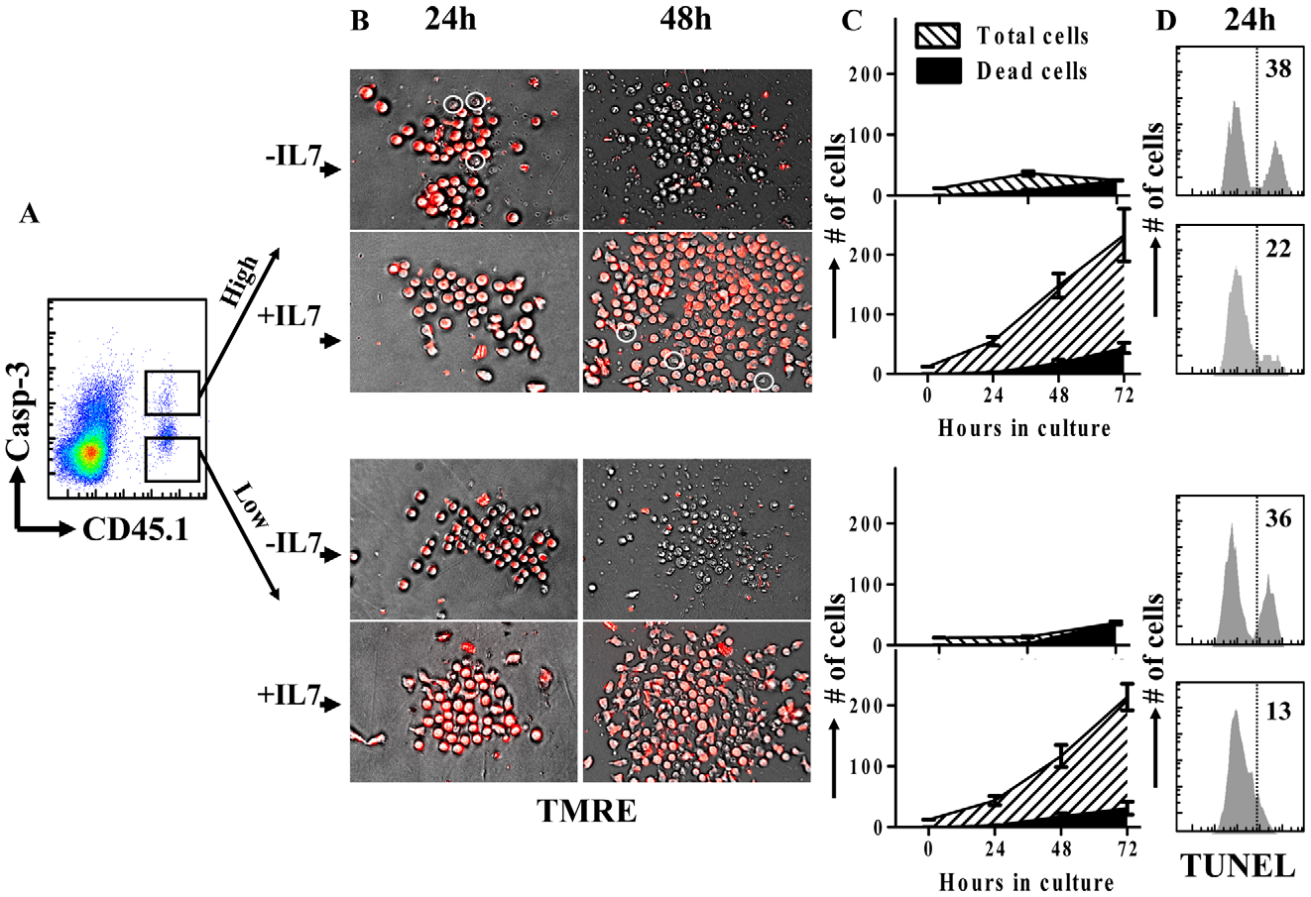
The majority of active caspase-3^hi^ cells do not progress to cell death during early antigen induced proliferation *in vivo*. Mice were parked with 10^4^ CD45.1^+^ OT1 spleen cells and challenged with 10^4^ LM-OVA (iv). Mice were sacrificed at day 5 post LM-OVA infection and CD8^+^ T cells were magnetically isolated by negative selection. Cells were stained with anti-CD45.1 antibody and PhiPhiLux. (A) Cells were sorted into CD45.1^+^ caspase-3 activity high and CD45.1^+^ caspase-3 activity low fractions. (B) Sorted cells were then plated at low cell densities (∼12.5 cells/well) and examined for viability during proliferation over several days using TMRE staining. (C) The total number and number of dead cells were counted directly in at least 6 replicate wells and the changes observed over 72 hours in the plates. (D) Cells were also maintained at higher concentrations (∼100 000/well) and stained for DNA cleavage (TUNEL) after 1 day in culture.

The lack of terminal cell death *in vivo* may be alternatively explained by the rapid clearance of dead cells by macrophages, thus we used the fluorogenic caspase-3 substrate PhiPhiLux to purify OVA-specific CD8^+^ T-cells at day 5 of infection into active caspase-3^hi^ and caspase-3^low^ fractions (Fig. 5A). In order to examine their viability directly over several days we utilized TMRE staining for mitochondrial activity (Fig. 5B, C). In the presence of the supportive cytokine (IL-7), active caspase-3^hi^ cells were able to persist and proliferate with a low level of concurrent cell death similar to active caspase-3^low^ cells. Conversely, when cells were placed in media without IL-7, both active caspase-3^hi^ and caspase-3^low^ cells died rapidly, underlining the necessity for IL-7 in proliferating CD8^+^ cell populations [24]. The viability of both caspase-3^hi^ and caspase-3^low^ populations was also confirmed by TUNEL staining at day 1 in culture (Fig. 5D). It should be noted that IL-7 did not inhibit apoptosis of CD8^+^ T cells induced by staurosporine (Figure S4.). Cells were seeded at very low numbers (12.5 cells/well) in order to avoid the statistical possibility that a minor subpopulation can completely overtake dying caspase3^hi^ cells. A hypothetical 10% contaminant subpopulation in the sorted caspase3^hi^ population would have to proliferate more than 50-fold (∼5.5 clonal divisions) in 24 h to overtake the culture completely; a highly unlikely scenario given the calculated rates of division of CD8^+^ T cells during their peak proliferative phase (∼3 divisions) [3], [25]. Rather, this data indicates that the induction of active caspase-3 associated with antigen induced proliferation of CD8^+^ T cells *in vivo* does not lead to significant cell death.

### Differentiated effector phenotype cells express basal levels of active caspase-3

**Figure 6:**
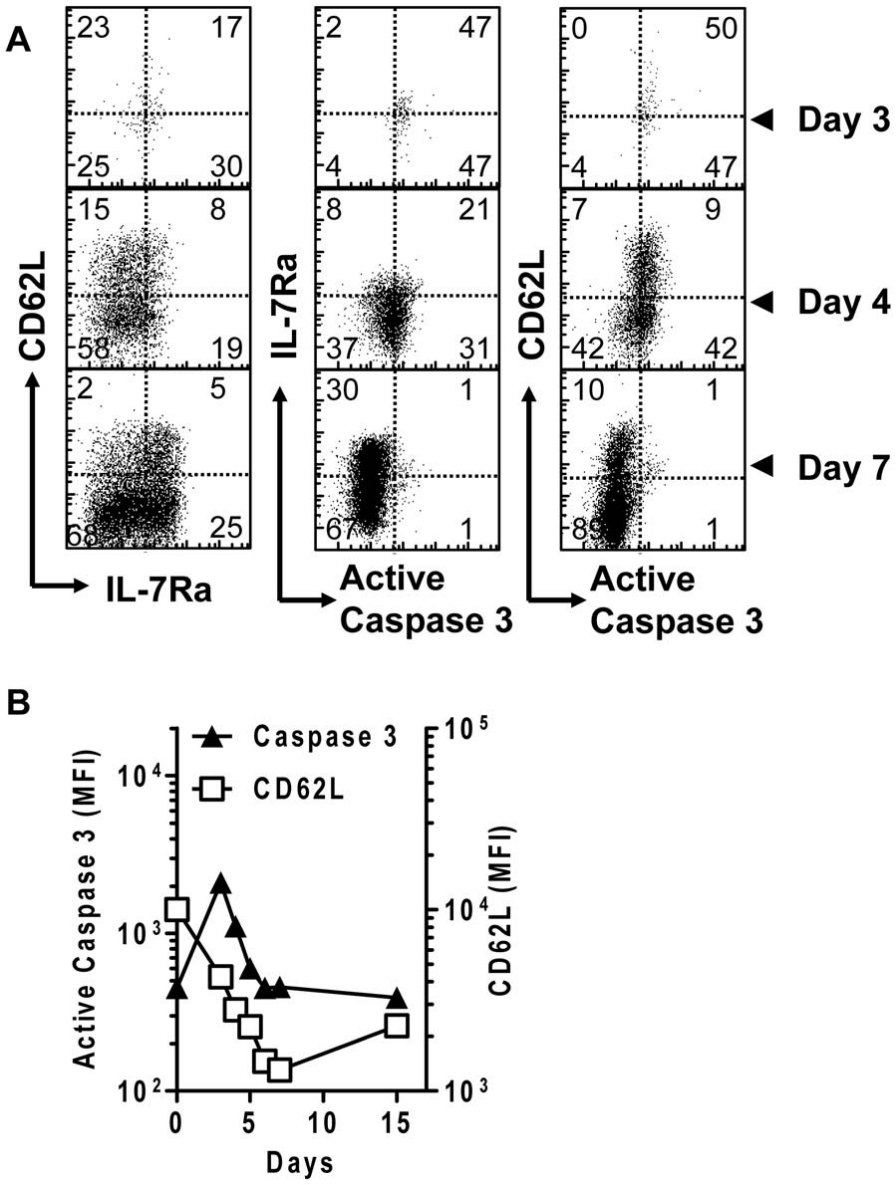
The activation of caspase-3 occurs in early activated cells before the emergence of fully differentiated effector cells. C57BL/6 mice were initially parked with 10^4^ OT1 spleen cells. After infection with 10^4^ LM-OVA, mice were sacrificed at various time points and spleens were harvested. Single cell suspensions were co-stained for CD8^+^, OVA-Tetramer, CD62L, IL7Rα and active caspase-3. (A) Scatter plots show the relative staining of OVA-tetramer^+^ CD8^+^ cells for activation markers and active caspase-3. Data is representative for at least 3 mice per time point. (B) OVA-tetramer^+^ CD8^+^ T cells were examined for active intracellular active caspase-3 and surface CD62L between days 0–15 of LM-OVA infection. (data is representative for 3 mice per timepoint).

Next, we wanted to assess more precisely when and how caspase-3 is up-regulated relative to T cell activation and proliferation. CD62L and IL-7Rα expression can be used to differentiate between naïve and differentiated effector CD8^+^ T cells, as well as the type of memory cells produced. Thus, we co-stained cells for CD62L, IL-7Rα and active caspase-3 over several time points. As naïve CD8^+^ T cells underwent differentiation, they initially showed high expression of active caspase-3, but few cells displayed an effector (CD62L^low^ IL-7Rα^low^) phenotype (Fig. 6A, B). At the peak of caspase-3 expression (day 3), CD62L expression had decreased only modestly. Subsequently, the expression of both active caspase-3 and CD62L decreased precipitously. At day 7, when the primed CD8^+^ T cells displayed mainly effector (CD62L^low^) phenotype, cells also expressed basal level of caspase-3. All memory subtypes in the later phase of the response showed low levels of active caspase-3. Thus, the timing of elevated caspase-3 activity matches the timing of CD8^+^ T cell proliferation, and precedes their differentiation to a primarily effector population.

**Figure 7:**
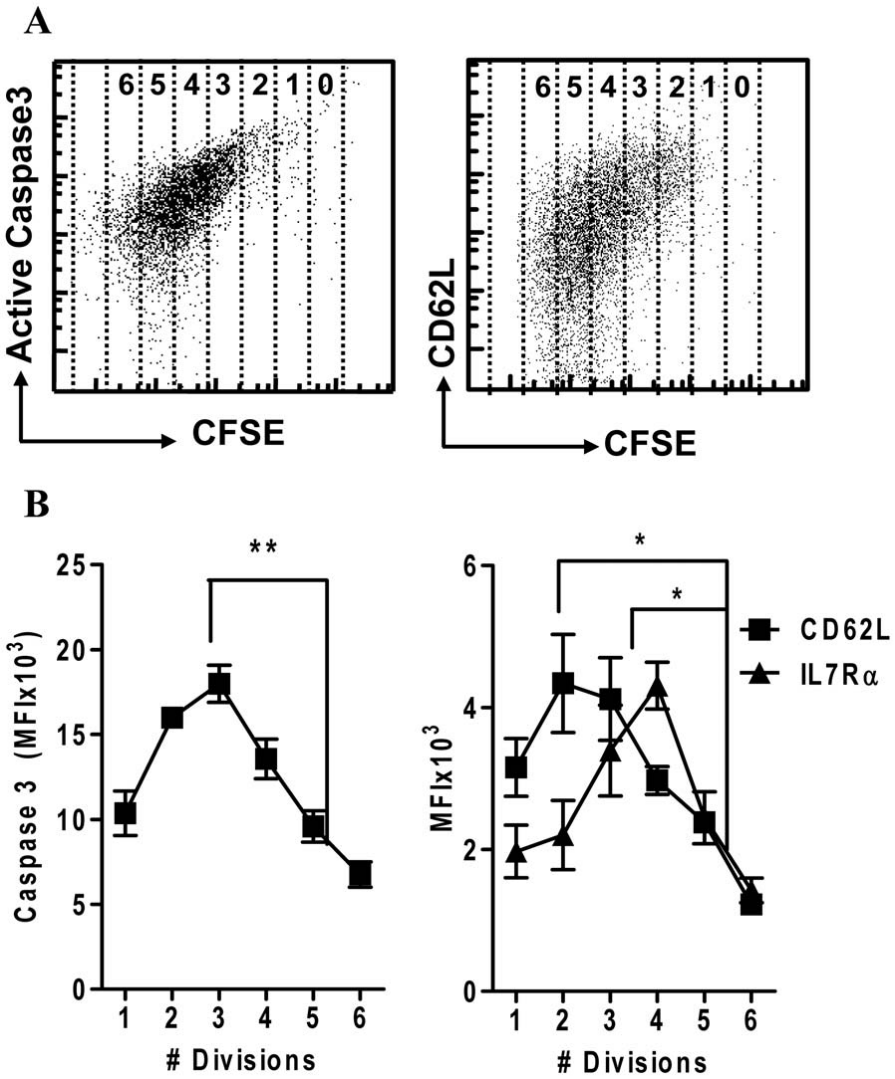
Caspase 3 and CD62L expression is progressively lost as proliferation proceeds. 10^4^ OT1 splenocytes were stained with CFSE and injected in C57BL/6 recipient mice concurrently with LM-OVA challenge. After 3, 4 and 5 days, mice were sacrificed (3 per time point) and spleen cells stained for expression markers. (A) Scatterplots show expression of active caspase-3 versus CFSE and CD62L in OVA-tetramer^+^ CD8^+^ T cells after 4 days in vivo. (B) OVA specific CD8^+^ T cells were gated for division number based on CFSE dilution. Cells show a significant, coordinate decrease in the expression of active caspase-3 and CD62L/IL7Rα as cell proliferation proceeds (***P<0.005, *P<0.05, n = 3 per timepoint).

To directly examine the mode of caspase-3 activation relative to their proliferation and differentiation, mice were concurrently injected with CFSE stained OT-1 CD8^+^ T cells and LM-OVA. This allowed the direct observation of proliferation versus the expression of active caspase-3 and differentiation markers at a single time point. Cells that had undergone only a few rounds of division expressed the highest levels of caspase-3, and this was progressively lost as the cells underwent further rounds of division and effector phenotype cells emerged (Fig. 7A, B). These results further support a model wherein caspase-3 is upregulated transiently during the proliferation of recently activated CD8^+^ T cells, and this apoptotic-like phenotype is progressively lost as cells proliferate further, and differentiated effector cells emerge.

### Caspase-3 activation occurs in lymphoid tissue

**Figure 8:**
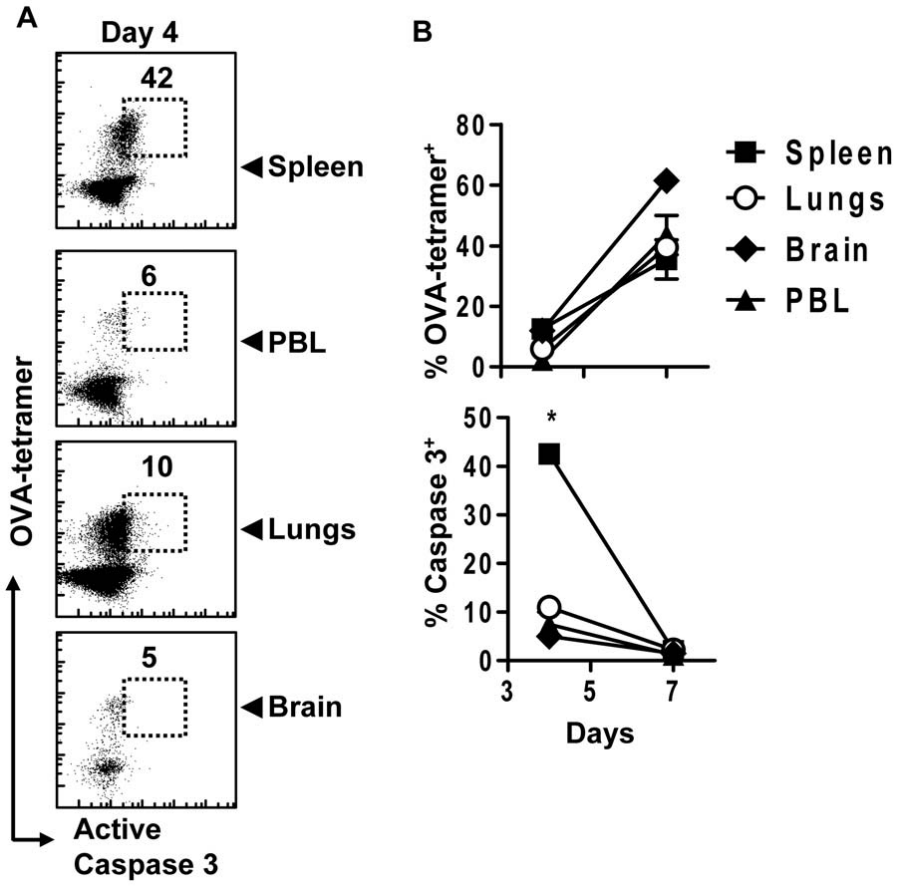
Elevated active caspase-3 is found primarily in secondary lymphoid tissues early in T cell activation. Mice parked with 10^4^ OT1 cells were challenged with 10^4^ LM-OVA. At various time points, mice were perfused and organs removed. Single cell suspensions were stained and analyzed by FACS. (A) Scatterplots show OVA-tetramer binding versus active caspase-3 expression in CD8^+^ T cells. Plots are representative of 3 mice analyzed. (B) The % of OVA-tetramer^+^ cells in each organ (upper right) and the change in the level of active caspase-3 expression in OVA specific CD8^+^ T cells (lower right) from each organ is shown. There is a significantly higher proportion of active caspase-3 high cells in the spleen when compared to PBL, lungs or brain at day 4 (*P<0.005, n = 2 per timepoint).

CD8^+^ T cell activation and proliferation occurs mainly within secondary lymphoid tissue such as spleen. Non-lymphoid tissue, such as lungs, can be exposed to numerous infectious agents that may influence the apoptotic phenotype, or caspase-3 expression in antigen-specific CD8^+^ T cells. We thus characterized the tissue localization of active caspase-3 during the response to LM-OVA. Splenic OVA specific CD8^+^ T cells showed elevated active caspase-3 at day 4 after LM-OVA infection. In contrast, active caspase-3 was minimally expressed in OVA-specific CD8^+^ T cells isolated from peripheral blood, lungs or brain (Fig. 8). A basal level of caspase-3 activity was observed in all tissues at day 7 after response. Thus the activation of CD8^+^ T cells drives the upregulation of active caspase-3, primarily within lymphatic tissue. Furthermore, differentiated effector cells that move into the periphery have only basal caspase-3 levels.

### Caspase-3 activation is determined by timing and strength of antigen-presentation

**Figure 9:**
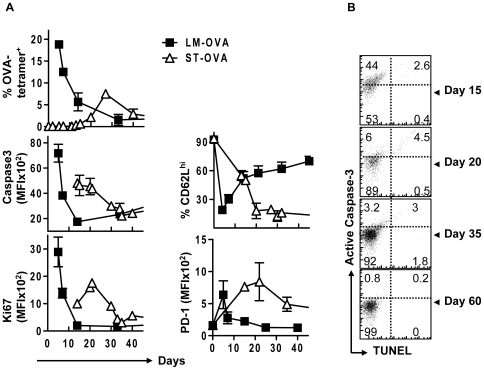
ST-OVA infection induces a lower and protracted increase in the expression of active caspase-3 consistent with delayed and muted antigen induced CD8^+^ T cell proliferation. B6129F1 mice that resist infection with ST were parked with 10^4^ OT1 cells and challenged with either 10^3^ LM-OVA or 10^3^ ST-OVA iv and sacrificed at various time points after infection. Single cell suspensions were obtained from spleens and stained with various markers indicated in the figure. Relative expression of various markers on OVA-tetramer^+^ CD8^+^ T cells was evaluated (A). Cells were also co-stained for DNA cleavage (TUNEL) vs. active caspase-3 expression (B). (n≥3 per timepoint).

While the LM-OVA model showed a clear correlation between the timing of early CD8^+^ T cell activation and upregulation of caspase-3 activity, we were interested in determining the expression of caspase-3 during another infection model that differed in the degree and timing of antigen-presentation. We have previously shown that antigen-presentation is highly muted and delayed during infection of mice with ST-OVA, resulting in the development of a CD8^+^ T cell response that peaks ∼day 20 [26]–[28]. Using OT-1 adoptive transfer and ST-OVA infection, we observed delayed and reduced induction of active caspase-3 coordinate with delayed and lower OVA-specific CD8^+^ expansion, relative to LM-OVA (Fig. 9A). Relatively muted and protracted caspase-3 activation corresponded directly with the timing of elevated Ki67, PD1 and CD62L expression. At around day 25–30 post infection (peak of response), cells maintained only a basal level of active caspase-3 and Ki67. During ST-OVA infection, the majority of primed cells display a prolonged effector/effector memory phenotype [26], [27], [29] as revealed by persistently low CD62L expression (Fig. 9A). However, similar to LM-OVA infection, the expression of active caspase-3 was not maintained on effector CD8^+^ T cells. Taken together, in both LM-OVA and ST-OVA infection models, caspase-3 was induced during the phase of active CD8^+^ T cell proliferation. Furthermore, active caspase-3 remained at basal levels as CD8^+^ T cell responses peaked and effector phenotype cells emerged. TUNEL data again indicates that the observed changes in the level of active caspase-3 do not correlate with significant cell death (Fig 9B).

### Caspase-3 cleavage is driven primarily by antigen *in vivo*

**Figure 10:**
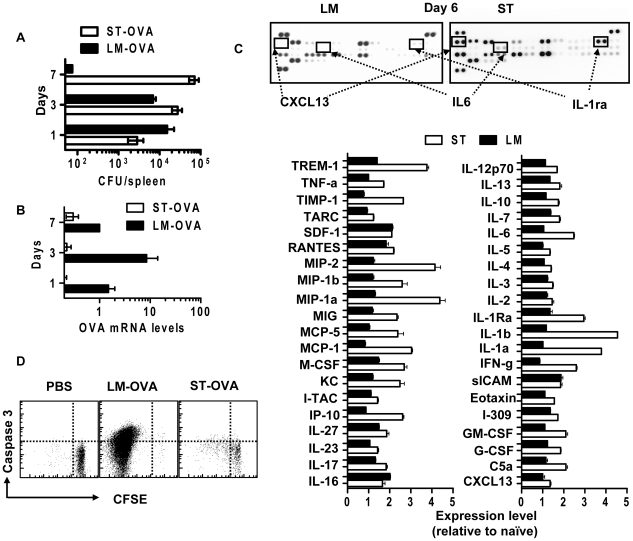
Caspase-3 is induced by antigen, not inflammation *in vivo*. B6129F1 mice were parked with 10^4^ OT1 spleen cells, then infected with ST-OVA or LM-OVA (10^3^, iv) and sacrificed at several time points between days 4 and 60 post-infection. Splenic suspensions were analyzed for (A) bacterial load by CFU, (B) bacterial OVA mRNA expression by qRT-PCR, and (C) the relative induction of 40 cytokines/chemokines by cytokine proteome array (day 6). (D) CFSE labeled OT1 cells were injected into B6129F1 mice that were challenged with LM-OVA or ST-OVA, and the proliferation and caspase 3 expression of OVA-specific evaluated at day 5 after infection. Scatter plots show data obtained from gated OVA specific CD8^+^ T cells. Data shown is representative for at least 3 mice per timepoint.

We next evaluated the relative influence of antigen versus inflammation on the induction of caspase-3 in CD8^+^ T cells *in vivo*. To address this, we resorted to comparing the two infection models, LM-OVA and ST-OVA and their relative timing and strength of active caspase-3 induction. Firstly, we measured the bacterial burden during the first seven days of infection. LM-OVA was rapidly cleared after the first few days of infection, whereas ST-OVA burden continued to increase over the seven day period (Fig. 10A). We also examined the relative level of OVA mRNA by qRT-PCR during infection of mice with LM-OVA and ST-OVA. While the spleens of mice infected with LM-OVA showed a significant peak in OVA transcription at day 3 post-infection, spleens of ST-OVA infected mice showed very little OVA mRNA by day 7 post-infection (Fig. 10B). This result explains why the CD8^+^ T cell response is delayed during infection of mice with ST-OVA [28], and links the timing of caspase-3 cleavage to the timing of antigen presentation. Next, we used a cytokine array to measure the expression of 40 cytokines/chemokines simultaneously in the spleens of LM-OVA and ST-OVA infected mice at day 6 post-infection. ST-OVA infection resulted in much higher expression of inflammatory cytokines/chemokines relative to LM-OVA (Fig. 10C). Taken together these data indicate LM-OVA infection induces more antigen load and more caspase-3 activation, whereas during ST-OVA infection, poor antigen load but higher inflammation fails to drive the upregulation of caspase-3 early on.

We also injected CFSE labeled OT-1 cells concurrently with LM-OVA and ST-OVA infection, and evaluated the proliferation of transferred OT-1 cells after 5 days *in vivo*. The majority of OT-1 cells in LM-OVA infected mice had reduced CFSE expression and higher levels of active caspase-3 (Fig. 10D), whereas OT-1 cells in ST-OVA infected mice remained mainly CFSE^hi^ and caspase3^low^. Thus, ST-OVA infection which induces massive inflammation, but little antigen expression results in poor cycling of CD8^+^ T cells, and does not influence caspase-3 expression in CD8^+^ T cells during the first week of infection. We also examined the effects of antigen versus inflammatory stimulation on active caspase-3 expression and cell cycling *in vitro*. While antigen stimulation can simultaneously increase cell proliferation (Ki67^hi^) and active caspase-3 expression, inflammatory stimulation in the form of heat killed bacteria (LM or ST) has little effect on caspase-3 expression in stimulated or unstimulated cultures (Figure S5). Taken together, these results indicate that antigen is the principal inducer of caspase-3 cleavage in CD8^+^ T cells.

### Caspase-3 is not activated during homeostatic proliferation

**Figure 11:**
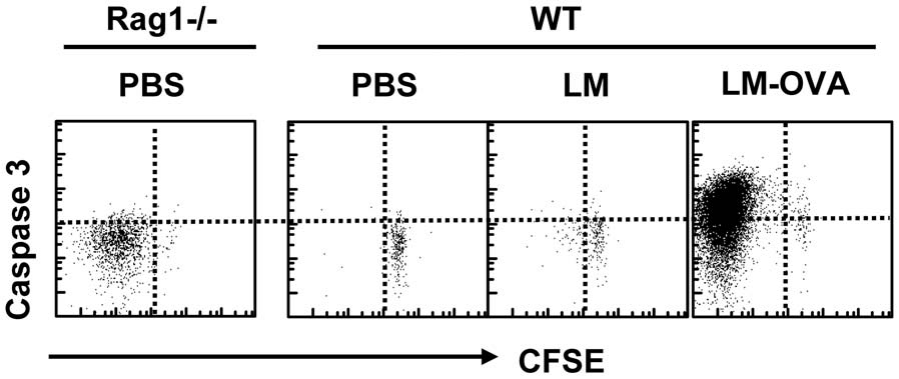
Caspase-3 activation does not occur during rapid homeostatic proliferation in vivo. Rag1-/- mice were injected with 10^4^ CFSE stained OT1 cells. WT mice were injected with 10^5^ CFSE stained OT1 cells concurrent with PBS, LM or LM-OVA (10^4^, iv). Four days later mice were sacrificed and spleen cell suspensions stained with anti-CD8 antibody, OVA-tetramer, followed by intracellular staining with anti-caspase-3 antibody. Scatterplots show data obtained from gated OVA specific CD8^+^ T cells and is representative of 3 mice per group.

Having noted that antigen stimulated proliferating CD8^+^ T cells express caspase-3, we determined whether non-antigen induced homeostatic CD8^+^ T cells proliferation is also associated with caspase-3 activation. The transfer of OT-1 CD8^+^ T cells to lymphopenic hosts (Rag1-deficient recipient mice) results in a relatively rapid proliferation of transferred CD8^+^ T cells in the absence of antigen. We transferred 10^5^ CFSE stained OT-1 CD8^+^ T cells to WT and Rag1-deficient mice. Groups of WT mice were also challenged with LM or LM-OVA. Spleens were removed at day 5 post-infection and cells were stained to evaluate the donor CD8 population and the expression of active caspase-3. In Rag-1-deficient hosts, transferred CD8^+^ T cells had undergone significant homeostatic proliferation as revealed by CFSE dilution, however, there was no apparent upregulation of caspase-3 activity (Fig. 11). Conversely, in the WT mice, only those mice challenged with LM-OVA showed proliferation associated with significant activation of caspase-3. Wild-type LM (without OVA) induced low level non-specific proliferation of transferred cells but failed to induce the expression of caspase-3 on OVA-specific CD8^+^ T cells. These results further indicate that caspase 3 is specifically induced by antigen presentation and not upregulated during non-specific or homeostatic proliferation.

## Discussion

CD8^+^ T cell responses can provide effective pathogen clearance, as well as long-term protection against pathogens due to their ability to develop into memory cells, however, the mechanisms that govern the highly dynamic processes of proliferation, contraction and memory formation are unclear [3]. In particular, the role of apoptotic mechanisms in proliferation [9], [10] and contraction [30] of primed CD8^+^ T cells remains elusive. Caspase-3 cleavage is often referred to as the key downstream apoptotic event, and can be activated by a variety of pathways including death receptor driven caspase-8 cleavage, or mitochondrial driven caspase-9 cleavage [31]. In this study we have used two divergent infection models to evaluate the influence of antigen and inflammation on the timing and duration of caspase-3 activation in antigen-specific CD8^+^ T cells during their proliferation, differentiation and contraction.

Our data clearly shows that caspase-3 is activated following antigen specific stimulation of CD8^+^ T cells. This adds to previous findings showing specific induction of caspase-3 mRNA following TCR stimulation [32]. An important question that remains is how caspase-3 cleavage might be induced following TCR engagement? Work in photoreceptors has shown that elevated intracellular calcium can activate calpains that in turn cleave caspase-3 [33]. Calpains lyse particular protein targets and have been proposed to stimulate the mitochondrial release of cytochrome C and activation of caspase-9 [34]. Similarly, high level calcium signaling associated with TCR stimulation [35] may lead to caspase-3 activation during CD8^+^ T cell stimulation.

It has recently been shown that caspase-3 activation occurs in T cells under anergizing conditions. Caspase-3 was shown to drive anergy via the cleavage of TCR signaling proteins Vav1 and Grb2 [36], and the downregulation of CD28 expression [37]. These results were obtained with T cell lines stimulated *in vitro* and it was not clear whether caspase-3 activation was also associated with normal CD8^+^ T cell activation. In contrast, our results (*in vitro* and *in vivo*) indicate that caspase-3 activation occurs during the ‘normal’ antigen specific activation of T cells. Indeed, active caspase-3 was consistently and directly associated with rapidly proliferating (Ki-67^+^) CD8^+^ T cells.

In a mouse model of *Lymphocytic choriomeningitis virus* (LCMV) infection wherein caspase-3 was also observed to be elevated during proliferative phases, it was suggested that pro-apoptotic mediators may function to degrade anti-apoptotic mediators and prepare cells for delayed apoptosis [38]. However, a critical question that remained was why the levels of downstream apoptotic mediators dropped to background levels before the onset of contraction. In addition, while short-lived effector cells (SLEC) that emerge at the peak of CD8^+^ T cell response (day 7) against LCMV express KLRG1 [39], we do not observe a strong correlation between active caspase-3 and KLRG1 expression in LM-OVA infection (data not shown). Thus, caspase-3 activation occurs transiently during the rapid phase of proliferation and does not appear to influence the differentiation of CD8^+^ cells to memory or effector phenotypes.

While early inflammation is an important determinant of CD8^+^ T cell contraction [30], [40], no key mechanisms have come to light that might explain the massive and rapid contraction of CD8^+^ T cell response after priming. Contraction proceeds normally in various hosts that are deficient in key apoptotic mechanisms [3], [41]–[43]. The death of staphylococcal enterotoxin B (SEB) stimulated T cells was shown to be mediated by proapoptotic Bcl-2 family member, Bim [44]. In the LCMV infection model, contraction of CD8^+^ T cell response was shown to be reduced, but not absent in Bim-deficient mice [7]. The precise evaluation of the extent of contraction is also complicated by the massive trafficking of CD8^+^ T cells to numerous non-lymphoid organs [45]. Our results indicate that by day 7 of LM-OVA infection, when effector-phenotype cells begin to contract, they express little of the apoptotic markers we examined. In agreement with this, it has previously been shown that the application of a caspase inhibitor has no effect on contraction of CD8^+^ T cell response [46]. These findings seem to solidify the conclusion that contraction does not occur by caspase 3 dependent apoptotic cell death.

Despite previous evidence that activation-associated caspase-3 induction does not drive the death of cells *in vitro* (18, 19), the prospect that a vigorous outgrowth of small population of caspase-3^low^ cells might mask a significant level of dying caspase3^hi^ cells remained a possibility. Thus, we examined proliferating cells directly at very low cell numbers, eliminating the possibility of a minority contaminating population being responsible for significant outgrowth. We also confirmed these findings *in vivo*, where despite the relatively high level of active caspase-3, we were unable to detect a significant population of TUNEL^+^ CD8^+^ T cells. Even purified caspase-3^hi^ cells did not reveal significantly elevated cell death when compared to those cells with the lowest level of caspase-3. We conclude that caspase-3 activation does not result in a significant amount of cell death concurrent with CD8^+^ T cell proliferation.

Our data indicates that caspase-3 is not a determinant of CD8^+^ T cell survival. In both caspase 3^hi^ or caspase 3^low^ populations on days 4, 5 or 7 after LM-OVA infection, no significant differences were noted in the relative death of the two populations, with both being highly dependent on IL-7 stimulation. Indeed, expression of IL-7 has been shown to be critical in development of CD8^+^ T cell memory [24]. It remains pertinent to ask, why would the cells appear to go through a seemingly apoptotic program, but not proceed to death subsequently? Differential subcellular localization of caspase-3 in activated CD8^+^ T cells has been previously observed, and may explain the fact that cells expressing active caspase-3 do not proceed to cell death. Whereas it must translocate to the nucleus during apoptosis, caspase-3 appears to associate primarily with the cell membrane during T cell stimulation [36]. It has also been proposed that caspase may be more substrate selective when activated in CD8^+^ T cells, perhaps targeting proteins regulating cell cycling [47]. There may also be an important induction of anti-apoptotic mediators in activated CD8^+^ T cells. The Birc (or IAP) proteins are classical cellular inhibitors of caspases, which bind to the active site of caspase and inhibit caspase activity [48]. Birc5 (survivin) has been shown to be highly upregulated by CD28 and OX40 signaling during CD8^+^ activation, and may be important in preventing caspase-3 mediated cell death [49], [50].

At day 3 after LM-OVA infection, when caspase-3 expression was at its peak, nearly all caspase-3^hi^ cells were also PD-1^hi^ and Annexin V^hi^. While the externalization of phosphatidyl serine (PS) on the surface of cells is considered a canonical marker of cell death, this generalization has been widely called into question. Externalization of PS in a non-apoptotic context has been observed after engagement of immune-receptors of mast cells, B cells and CD8^+^ T cells [20], [51], [52]. Similarly, PD-1 has been shown to be a marker of recent antigenic exposure as well as exhaustion in CD8^+^ T cells [53]. The connection of PD-1 to apoptotic mechanisms in CD8^+^ T cells remains unclear [54]. While the co-expression of canonical apoptotic mediators seems to point to significant cell death in the activated CD8^+^ T cell population, this is inconsistent with the timing of the onset of contraction, and our cell viability observations.

Classically, apoptotic mechanisms enact programmed cell death, although they are increasingly being identified in roles outside of cell death. A new paradigm regards apoptotic pathways as complex signaling mechanisms that interact with a wide range of cellular pathways and will not always trigger cell death, as has been the subject of a number of recent reviews [10], [55], [56]. Caspase-3 cleavage specifically, has recently been shown to be vital to the normal differentiation of skeletal muscle cells [57], macrophages [58], erythroblasts [59], as well as others. Caspase activity has even been observed to prevent necrotic cell death in immortalized cell lines [60]. Similarly, regulation of cell death by caspases may facilitate T cell viability and expansion [61]. Indeed, the general inhibition of caspase activity has been shown to limit the proliferation of T cells *in vitro* [17] (Figure S6). While, the broad range of caspase-3 targets makes it difficult to dissect the exact mechanisms by which caspase-3 affects the proliferation of CD8^+^ T cells, this may in fact be a key functional property in T cell activation. The ability of apoptotic proteases, such as caspase-3, to target many protein signaling networks [62] may make them well suited tools to precipitate change in cellular signaling program from a naïve latent state to one of active proliferation and differentiation.

Thus, using two different infection models that differ in the relative expression of antigen versus inflammation, we have shown that TCR signaling, and not inflammation, is the key mechanism that drives the induction of caspase-3. Taken together these results resolve some controversies by providing strong evidence that active caspase-3 is: (a) directly stimulated by antigen, (b) expressed transiently during proliferation, (c) not associated with contraction of the response, (d) coupled with activation of other canonical apoptotic mediators, and (e) does not lead to cell death.

## Materials and Methods

### Mice and infections

All animals were housed in the animal facility of the Institute for Biological Science and maintained according to CCAC guidelines. Protocols and procedures (Protocol #2008-10) were approved and monitored by the National Research Council of Canada-Institute for Biological Sciences Animal Care and Ethics Board.

C57BL/6J mice, 3–4 weeks of age were obtained from Jackson Labs (Bar Harbor, ME). B6129F1 mice were generated in house in our animal facility by mating 129X1SvJ females with C57BL/6 males. Recombinant *Listeria monocytogenes* (LM) and *Salmonella enterica* serovar typhimurium (ST) expressing OVA have been described previously [26], [63]. OT-1 TCR transgenic mice (CD45.1^+^ or CD45.2^+^) were bred in house. Mice were injected first with 10^4^ OT-1 cells (intravenously) and challenged several days later intravenously with LM-OVA (10^4^) or ST-OVA (10^3^) in 0.9% NaCl.

### Isolation of cells from various organs

For the examination of CD8^+^ T cells in various organs, mice were first bled then anaesthetized and perfused before sacrificing using 50 mL of PBS. Following this, organs were removed (lungs, brain, spleen) and single cell suspensions were made. For lungs and brain, cells were resuspended in a 40% percol solution layered over 70% percol. After spinning, the lymphocyte fraction was collected from the 40% fraction. Cell staining was performed as described below.

### Antigen-presentation models

Antigen-presentation was evaluated using in vitro and in vivo models. For evaluation of antigen-presentation in vitro, a single cell suspension of OT-1 splenocytes were seeded in a 24 well plate at 5×10^6^ cells per ml of RPMI with 8% FBS. Soluble SIINFEKL (OVA_257–264_) peptide was added directly to the culture (0.1 pg to 1 µg). Cells were allowed to proliferate for 24–72 hours and then analyzed by FACS (as described below). In some cases OT-1 cells were first labeled with 1 µM of carboxyfluorescein succinimidyl ester (CFSE) for 8 minutes. After 3–4 days of culture, cells were harvested from the wells and stained and the expression of CFSE or other markers evaluated by flow cytometry. For evaluation of antigen-presentation in vivo, CFSE labeled OT-1 cells were injected (5×10^6^) into mice iv. Within 3–4 days, mice were infected with various bacteria through the iv route. Four-five days after infection, spleens were removed from the recipient mice. The presence of donor OT-1 CD8^+^ T cells (CD45.1^+^) and the reduction in CFSE intensity of donor cells was evaluated.

### Flow cytometry

Antibodies were obtained from BD Biosciences (Ontario, Canada). For surface staining, aliquots of cells (10×10^6^) were incubated in 100 µl PBS plus 1% BSA (PBS-BSA) with anti-CD16/32 at 4°C. After 10 min., cells were stained with PE-H-2K^b^OVA_257–264_ tetramer and various antibodies (anti-CD8, anti-CD62L, anti-PD-1, and/or anti- IL-7Rα) for 30 min. at room temperature. PE-H-2K^b^OVA_257–264_ tetramer was obtained from Beckman Coulter (Fullerton, California). Cells were washed, fixed in 0.5% formaldehyde and acquired on BD Biosciences FACS Canto analyzer.

For analysis of apoptosis, aliquots of cells (10×10^6^/ml) were surface stained initially with anti-CD8 antibody and OVA-tetramers as described above. This was followed by apoptotic staining as follows. For staining with Annexin V(BD – Cat#550474), cells were incubated with Annexin V (BD – Cat # 556419) for 15 minutes in 3 ml Annexin binding buffer (provided in Annexin staining kit). In order to stain cells for active caspase-3, cells were then washed, fix/permeabilized (BD – Cat #554714) and stained with a biotin conjugated antibody against active caspase-3 (BD - Cat#550557), before streptavidin labeling. In order to stain for the cleavage activity of caspase a cell-permeable fluorogenic substrate (PhiPhiLux – OncoImmunin Maryland, USA - Cat# A304R1G-3) was used according to the manufacturers recommended protocol. DNA cleavage was examined using the Apo-Direct kit (BD – Cat #556381). Briefly, cells were stained extracellularly, then fixed using a combination fixative/permeabilization solution (BD – Cat #554714). Cells were then stained with a solution containing terminal transferase enzyme and a FITC labeled dNTP. After incubation at 37°C for 1 hour cells were then fixed in 0.5% formaldehyde and read by flow cytometry. For cell death experiments, OT-1 cells were sometimes stimulated with 1 µg/ml of staurosporine for 6 hours to induce apoptosis.

### *In-vitro* LM-OVA activation, limiting dilution analysis and imaging

OT1 spleen cells were obtained from a naïve donor animal and placed in a flask at approximately 5×10^6^ cells/ml. 10^4^ LM-OVA bacteria were then added to the flask and the infected culture was incubated overnight at 37°C. At this time, cells were spun down and washed in fresh media to remove bacteria from the culture. A high amount of gentamicin (50 µg/ml) was also added to prevent further bacterial replication. Cells were then returned to a flask at 2×10^6^ cells/ml and allowed to proliferate overnight. At 48 hours after initial infection the cells are split to allow further proliferation to occur; at this time supporting IL-7 is also added to the culture at 1 ng/ml. After 3 days in culture, an aliquot of cells was stained using a similar procedure as detailed above to confirm that ≥90% of cells are CD8^+^ and active caspase-3^+^. Cells were then counted and placed in a 96 well round bottom flask at approximately 200 cells per well. Two fold dilutions were performed row-wise down to approximately 6.25 cells/well. At various time intervals, TMRE was added at 1 nM and live cells were examined using Olympus IX81 fluorescent microscope after 18 h. At least 6 wells were examined for every dilution of cells. 12.5 cells/well was chosen for daily analysis because the number of cells was adequate for counting, while providing a small enough number to preclude the possibility that a sub-population outgrowth accounts for the majority of the culture. Similarly, day 12 cells were examined for viability at low cell numbers.

### Cell sorting

Spleens were obtained from CD45.1^+^ OT1 parked CD45.2^+^ C57BL6/J recipient mice at day 5 after LM-OVA infection. CD8^+^ T cells were then enriched by negative selection (Stem cell Cat#19753) according to the manufacturer's instructions. Cells were stained for CD45.1 APC and caspase activity using PhiPhiLux. Cells were then sorted on MoFlo cell sorter into CD45.1^+^ OVA-specific CD8^+^ T cells that express low or high levels of caspase 3 activity. Sorted cells were then placed in 96 well plates with or without the addition of supporting IL-7 (1 ng/ml) using limiting dilutions. Cells were examined for viability over several days using TMRE staining similarly as described above.

### Cell imaging

Cells were stained similarly as described for FACS analysis. Cells were then resuspended in a small amount of Vecta Shield and mounted on slides. Slides were then examined on Olympus IX81 fluorescence microscope.

### Proteomic analysis

Infected mouse spleens were screened using the R&D Systems Proteome Profiler™ Mouse Cytokine Array Panel A Array Kit as per the manufacturer's instructions. In brief spleen samples were collected and homogenized in PBS containing 1% phenylmethanesulphonylfluoride (PMSF) and stored at −80°C until use on the array. Membranes were processed using the manufacturer's protocol and treated with Super signal West Femto chemiluminiscent detection reagent for 2–3 minutes. Images were captured by a Flourochem 8900 (Alpha Innotech) imager. Spot densitometry was measured using the AlphaEaseFC Software (Alpha Innotech) and each membrane was calibrated to a positive control standards. Values obtained from infected spleens were compared to naïve spleens and fold changes calculated.

### Quantitative RT-PCR

qRT-PCR was performed as described in detail previously [42], [64]. Expression of OVA mRNA was evaluated in LM-OVA and ST-OVA infected spleens *in vivo at* various timepoints. 5–10 µg of total RNA was taken for cDNA synthesis. After cycling, a melting curve protocol was performed to verify that the products had the expected melting temperature.

### Statistics

For the experiments listed above statistical analysis was performed using GraphPad Prism 5. Methods used included Pearson correlations to test similarity in expression patterns for many of the proteins examined. Those figures which describe significant correlation have been confirmed to have a Pearson r>0.95. Students T tests were also used to confirm significance of results.

## Supporting Information

Figure S1 **Active caspase-3 does not correlate with significant cell death in** ***in vitro*** **activated CD8^+^ OT1 T-cells.** OT1 spleen cells were placed in a single cell suspension and exposed overnight to 10^4^ LM-OVA bacteria. Bacteria were then washed from the culture and a high level of gentamicin was added to inhibit further bacterial growth. Cells were allowed a further 2 days for CD8^+^ T cells to proliferate. Cultures were then confirmed to contain >90% CD8^+^ OT1 T-cells and an aliquot was stained for active caspase-3 (A). Cells were then placed in 96 well round-bottom plates and diluted to 25 cells/well (B, top panels). Over several days, cells were examined for viability using TMRE (red colour in images) to mark metabolically active cells. Similarly, cells at 12 days after *in vitro* LM-OVA activation were evaluated for caspase-3 expression (A), and plated at 50 cells/well and examined for viability over several days (B, bottom panels). The total number and the number of dead cells were recorded for at least 6 replicate wells and tracked over 72 hours in the plate (C). Histograms show the level of active caspase-3 in day 3 and 12 *in vitro* activated T cells respectively (A).
(TIF)

Figure S2 **Caspase-3 activation is similar in adoptively transferred and endogenous CD8^+^ T cell response.** C57BL/6J mice were parked with 10^4^ CD45.1^+^ OT1 splenocytes or no cells at all, and then both groups were challenged with 10^4^ LM-OVA (iv). At days 5 and 9 post-infection, spleens were extracted and stained with anti-CD8, OVA-tetramer, anti-caspase-3 and, anti-Ki67 antibodies. Graphs show MFI of CD8^+^/OVA-tetramer^+^ populations for expression of (A) active caspase-3 and (B) Ki67 over time. Data shown is representative of at least 2 mice per time-point.
(TIF)

Figure S3 **Despite high caspase-3 activity, OVA-specific CD8^+^ T Cells obtained on day 4 of LM-OVA infection do not progress to DNA fragmentation and cell death over 24 hours** ***ex vivo*** **.** Recipient mice were parked with 10^4^ OT1 splenocytes and challenged with 10^4^ LM-OVA intravenously. At day 4 post infection, cells were removed and placed ex vivo in RPMI with 8% FBS for varying amounts of time. Apoptosis was induced in control cells by addition of 1 µg/ml staurosporine. Cells were then stained for (A) CD8, OVA-tetramer and active caspase-3 or (B) CD8, OVA-tetramer and DNA fragmentation (TUNEL). Plots show gated CD8^+^ T cell populations. Data is representative for 2 experiments.
(TIF)

Figure S4 **IL-7 does not rescue apoptotic control cells, induced using staurosporine.** Spleen cells were placed in culture at approximately 10^6^ cells/well in 24 well plates. Cells were then treated with IL-7 (1 ng/mL) and/or staurosporine. Control cells received no treatment. After 24 hours cells were stained for CD8^+^ expression and examined for viability by exclusion of propidium iodide.
(TIF)

Figure S5 ***In vitro*** **antigenic stimulation, but not inflammation, drives coordinated upregulation of cell cycling and caspase-3 activation.** OT-1 cells were placed in culture and either stimulated 1 µg/ml of SIINFEKL peptide or left unstimulated. Additional inflammatory stimulation was added to some cultures by adding (A) 10^4^ heat killed *Listeria monocytogenes* (HK-LM) or (B) 10^6^ heat killed *Salmonella* typhimurium. Scatterplots show the expression of caspase-3 versus Ki67 in gated CD8^+^ T cells.
(TIF)

Figure S6 **The specific inhibition of caspase-3 results in a significant reduction in the proliferation of CD8^+^ T cells in response to antigenic stimulation.** 10^5^ CFSE stained OT1 cells were stimulated in vitro with OVA peptide in the presence or absence of 50 µM caspase-3 inhibitor (z-DQMD-FMK) and examined for proliferation after 48 hours. There is a significant decrease in proliferation induced by OVA peptide in the presence of caspase-3 inhibitor (* P<0.05).
(TIF)
