# Supplementary figures and images for: Caspase-3 Is Transiently Activated without Cell Death during Early Antigen Driven Expansion of CD8+ T Cells In Vivo
Source: PLoS One. 2010 Dec 22;5(12):e15328. doi: 10.1371/journal.pone.0015328 (PMC3008739; doi:10.1371/journal.pone.0015328)

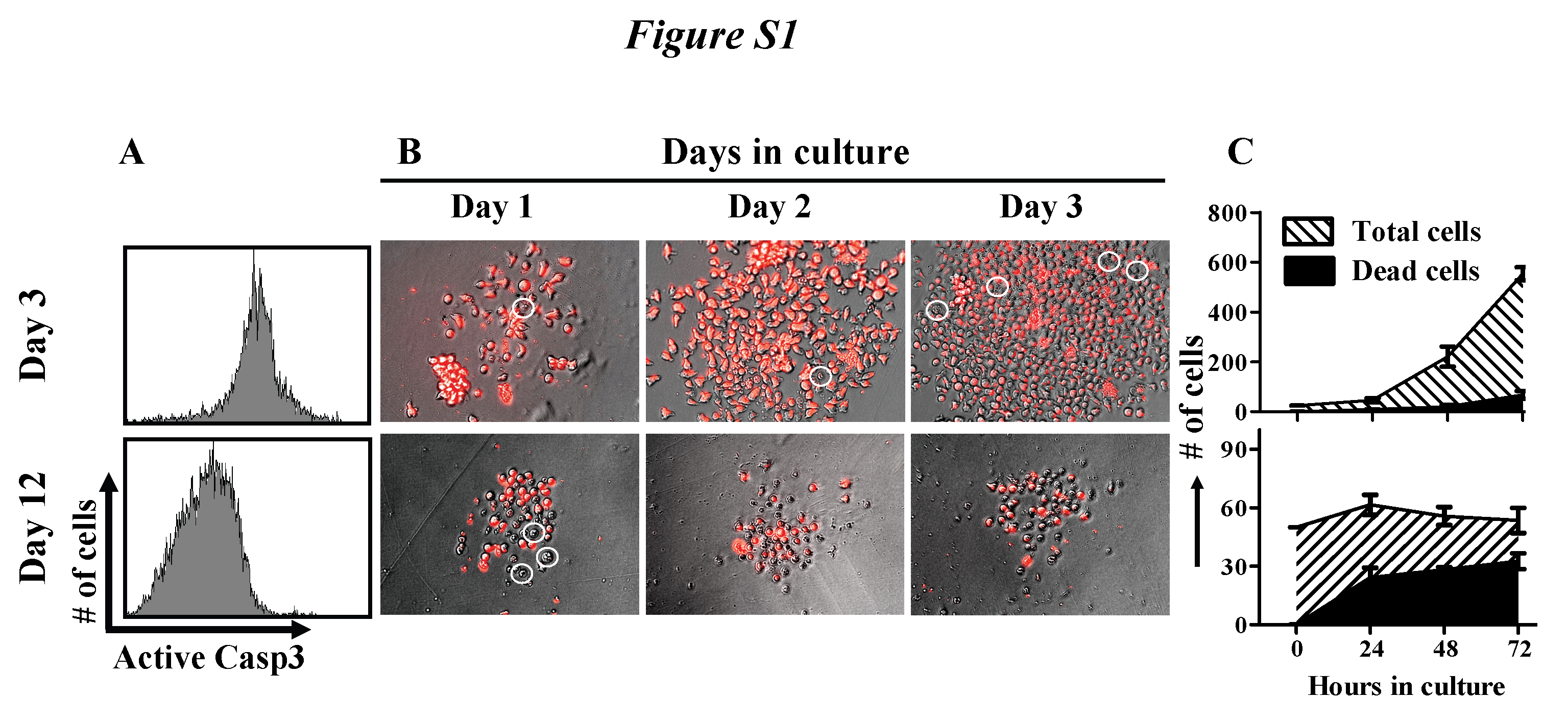

Supplement: Figure S1 — Active caspase-3 does not correlate with significant cell death in in vitro activated CD8+ OT1 T-cells. OT1 spleen cells were placed in a single cell suspension and exposed overnight to 104 LM-OVA bacteria. Bacteria were then washed from the culture and a high level of gentamicin was added to inhibit further bacterial growth. Cells were allowed a further 2 days for CD8+ T cells to proliferate. Cultures were then confirmed to contain >90% CD8+ OT1 T-cells and an aliquot was stained for active caspase-3 (A). Cells were then placed in 96 well round-bottom plates and diluted to 25 cells/well (B, top panels). Over several days, cells were examined for viability using TMRE (red colour in images) to mark metabolically active cells. Similarly, cells at 12 days after in vitro LM-OVA activation were evaluated for caspase-3 expression (A), and plated at 50 cells/well and examined for viability over several days (B, bottom panels). The total number and the number of dead cells were recorded for at least 6 replicate wells and tracked over 72 hours in the plate (C). Histograms show the level of active caspase-3 in day 3 and 12 in vitro activated T cells respectively (A). (TIF) [file pone.0015328.s001.tif]

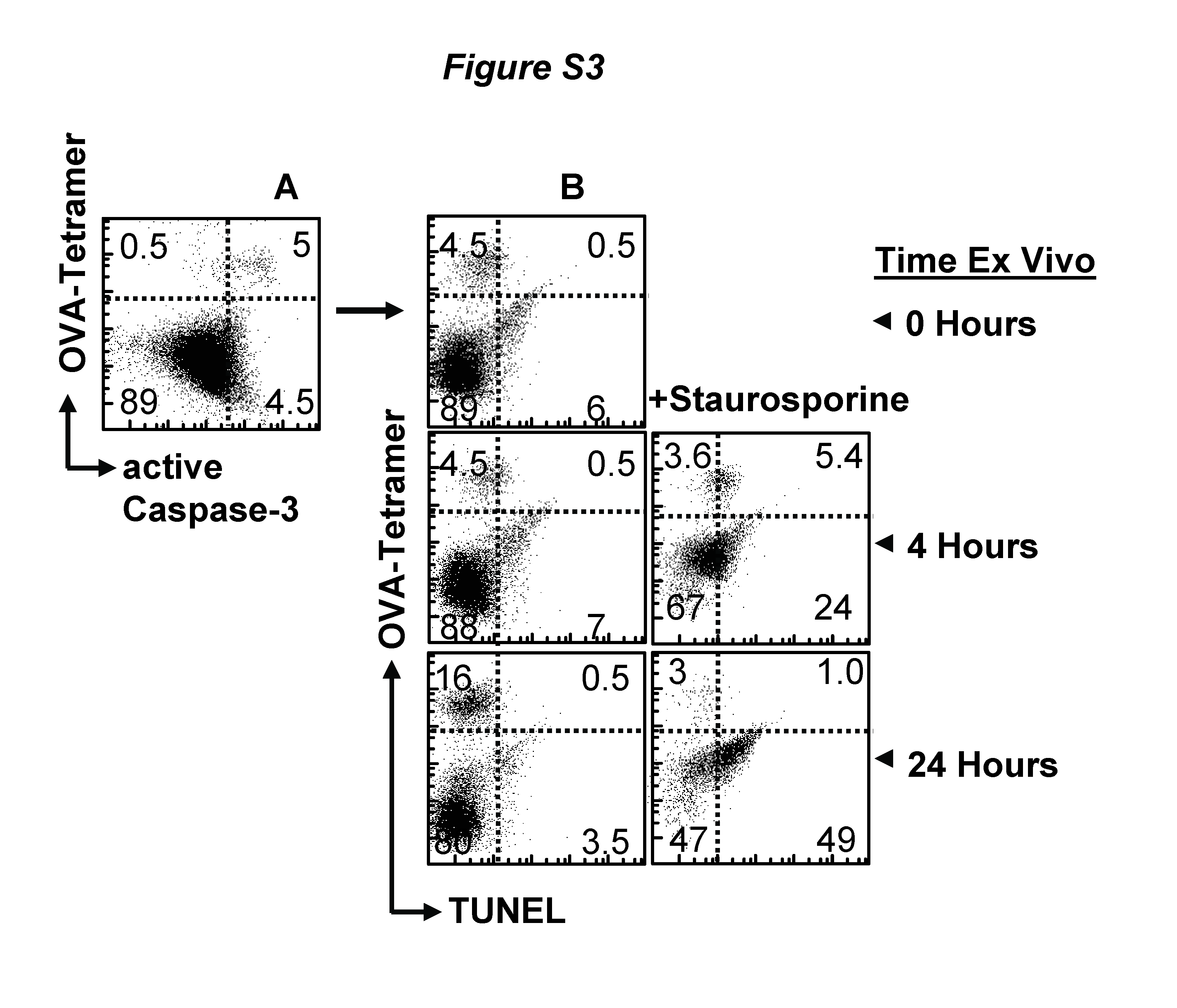

Supplement: Figure S3 — Despite high caspase-3 activity, OVA-specific CD8+ T Cells obtained on day 4 of LM-OVA infection do not progress to DNA fragmentation and cell death over 24 hours ex vivo . Recipient mice were parked with 104 OT1 splenocytes and challenged with 104 LM-OVA intravenously. At day 4 post infection, cells were removed and placed ex vivo in RPMI with 8% FBS for varying amounts of time. Apoptosis was induced in control cells by addition of 1 µg/ml staurosporine. Cells were then stained for (A) CD8, OVA-tetramer and active caspase-3 or (B) CD8, OVA-tetramer and DNA fragmentation (TUNEL). Plots show gated CD8+ T cell populations. Data is representative for 2 experiments. (TIF) [file pone.0015328.s003.tif]

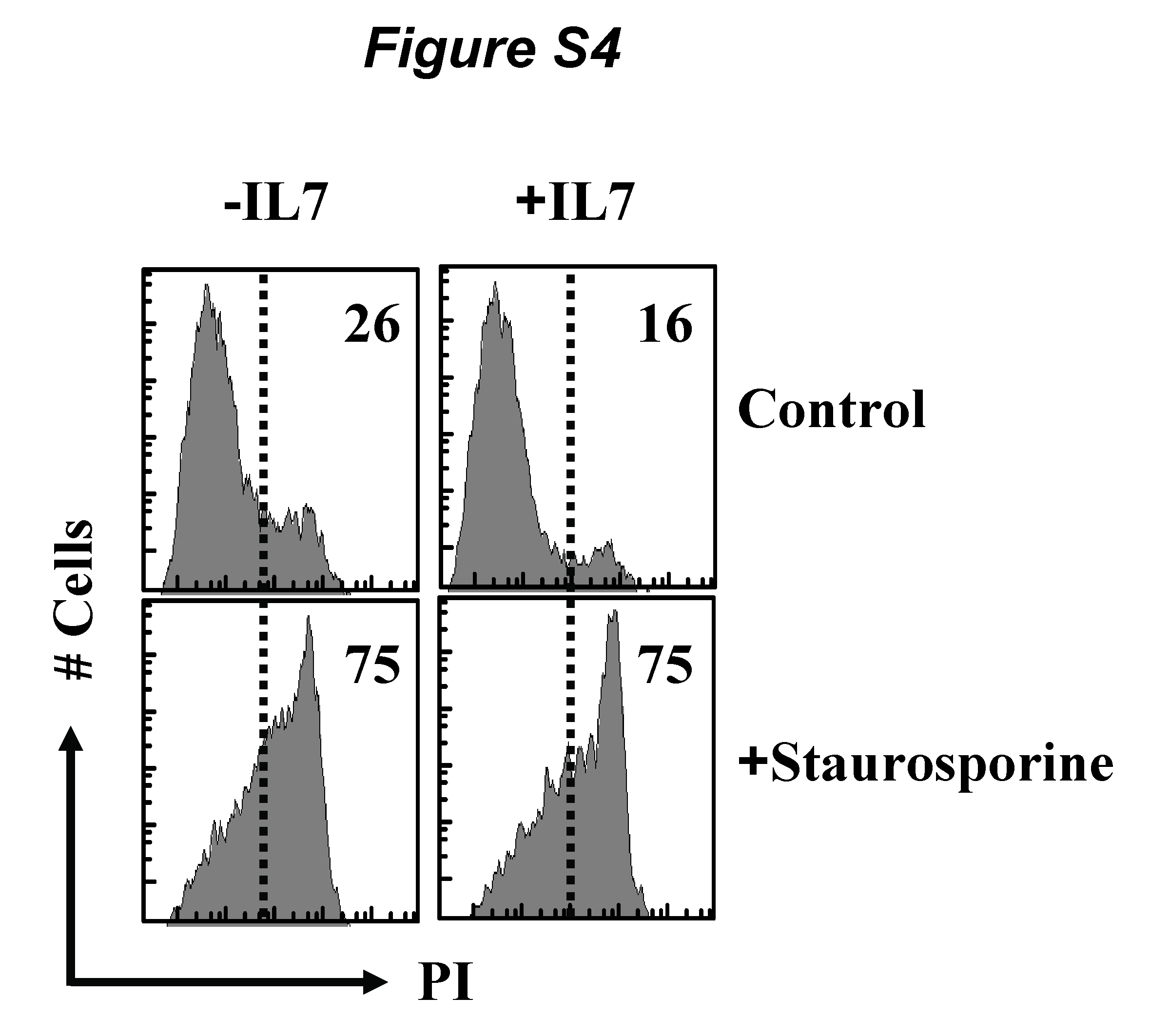

Supplement: Figure S4 — IL-7 does not rescue apoptotic control cells, induced using staurosporine. Spleen cells were placed in culture at approximately 106 cells/well in 24 well plates. Cells were then treated with IL-7 (1 ng/mL) and/or staurosporine. Control cells received no treatment. After 24 hours cells were stained for CD8+ expression and examined for viability by exclusion of propidium iodide. (TIF) [file pone.0015328.s004.tif]

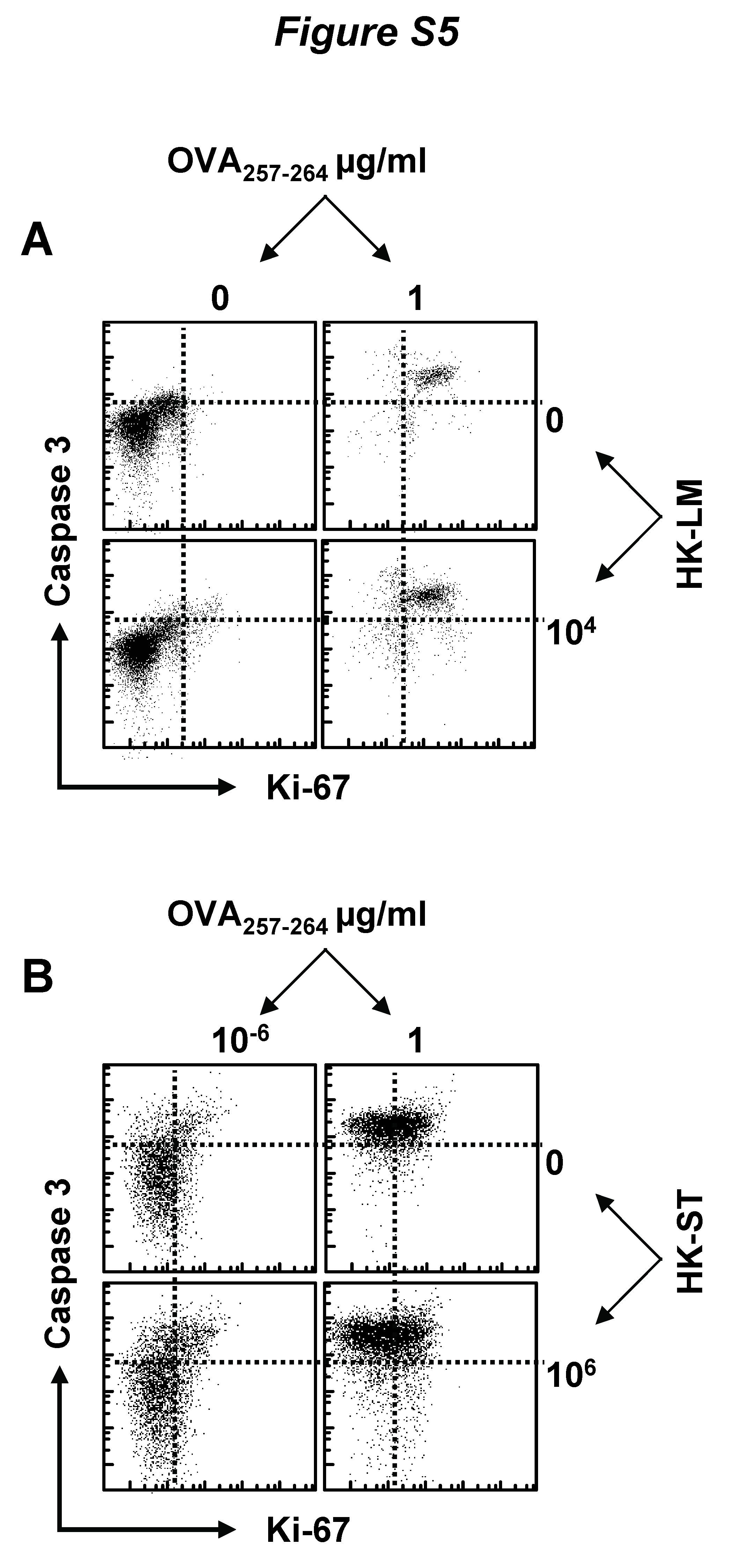

Supplement: Figure S5 — In vitro antigenic stimulation, but not inflammation, drives coordinated upregulation of cell cycling and caspase-3 activation. OT-1 cells were placed in culture and either stimulated 1 µg/ml of SIINFEKL peptide or left unstimulated. Additional inflammatory stimulation was added to some cultures by adding (A) 104 heat killed Listeria monocytogenes (HK-LM) or (B) 106 heat killed Salmonella typhimurium. Scatterplots show the expression of caspase-3 versus Ki67 in gated CD8+ T cells. (TIF) [file pone.0015328.s005.tif]
